# Defining Continuous Glucose Monitor Time in Range in a Large, Community-Based Cohort Without Diabetes

**DOI:** 10.1210/clinem/dgae626

**Published:** 2024-09-11

**Authors:** Nicole L Spartano, Naznin Sultana, Honghuang Lin, Huimin Cheng, Sophia Lu, David Fei, Joanne M Murabito, Maura E Walker, Howard A Wolpert, Devin W Steenkamp

**Affiliations:** Boston University's and National Heart, Lung, and Blood Institute's Framingham Heart Study, Framingham, MA 01702, USA; Section of Endocrinology, Diabetes, Nutrition, and Weight Management, Boston University Chobanian and Avedisian School of Medicine (BUCASM) and Boston Medical Center, Boston, MA 02118, USA; Department of Biostatistics, Boston University School of Public Health (BUSPH), Boston, MA 02118, USA; Department of Medicine, University of Massachusetts Chan Medical School, Worcester, MA 01655, USA; Department of Biostatistics, Boston University School of Public Health (BUSPH), Boston, MA 02118, USA; Department of Biostatistics, Boston University School of Public Health (BUSPH), Boston, MA 02118, USA; Boston University's and National Heart, Lung, and Blood Institute's Framingham Heart Study, Framingham, MA 01702, USA; Boston University's and National Heart, Lung, and Blood Institute's Framingham Heart Study, Framingham, MA 01702, USA; Section of General Internal Medicine, Department of Medicine, BUCASM and Boston Medical Center, Boston, MA 02118, USA; Boston University's and National Heart, Lung, and Blood Institute's Framingham Heart Study, Framingham, MA 01702, USA; Department of Health Sciences, Sargent College of Health and Rehabilitation Sciences, Boston University, Boston, MA 02215, USA; Section of Endocrinology, Diabetes, Nutrition, and Weight Management, Boston University Chobanian and Avedisian School of Medicine (BUCASM) and Boston Medical Center, Boston, MA 02118, USA; Section of Endocrinology, Diabetes, Nutrition, and Weight Management, Boston University Chobanian and Avedisian School of Medicine (BUCASM) and Boston Medical Center, Boston, MA 02118, USA

**Keywords:** continuous glucose monitoring, prediabetes, technology and diabetes, epidemiology, glycemic traits

## Abstract

**Context:**

Continuous glucose monitor (CGM) companies are beginning to market these sensors to populations without diabetes, but the range of CGM values clinicians should expect to see for this population is unclear because there have been no large studies reporting these ranges.

**Objective:**

This work aimed to report the physiological range of CGM time in range values observed across glycemic status, including individuals without diabetes, to serve as a reference for clinicians.

**Methods:**

The Framingham Heart Study, a prospective cohort study, was conducted among community-dwelling adults with normoglycemia (n = 560), prediabetes (n = 463), and diabetes (n = 152). We conducted a cross-sectional investigation in participants who wore a Dexcom G6 Pro CGM (in blinded mode) for 7 or more complete days. Main outcome measures included CGM metrics including mean glucose and time spent in glucose ranges.

**Results:**

Normoglycemic participants (mean age 58.5 years, 64.5% women, 93.3% non-Hispanic White) spent 87.0% time in the 70 to 140 mg/dL CGM range, and, on average, more than 15 minutes/day (1.2% time) at more than 180 mg/dL. Furthermore, normoglycemic participants spent approximately 3 hours/day (12.1% time) with CGM glucose at more than 140 mg/dL. On average, participants with prediabetes and diabetes spent 77.1% and 46.2% of time in the 70 to 140 mg/dL range, respectively.

**Conclusion:**

Our results contribute to the understanding of the physiological CGM range in more than 1000 participants without diabetes. These results are also important for clinicians to reference as CGM sensors become more widely accessible to people without known diabetes.

New continuous glucose monitor (CGM) sensors hit the wearables market this year (2024), such as Abbott's Libre Lingo and Dexcom's Stelo, that can be used by individuals without diabetes (1, 2). To date, there have been no large studies describing normative CGM time in range values in this population. Previous studies reporting normative CGM metrics in individuals without diabetes have been smaller (<100 adults), included only nonobese individuals, and asked participants to perform daily manual calibration using finger-stick blood glucose measurements (3). Many of the large CGM brands have made their CGM sensors factory-calibrated, removing the need for manual calibration, and making them more user-friendly to an individual who is not familiar with self-monitoring blood glucose.

Despite a lack of research showing that using CGM sensors improves health outcomes in individuals without diabetes, there seems to be a growing interest in CGM use in this population (4). We anticipate that there will be an increase in the number of CGM reports from patients without diabetes that health-care providers are asked to interpret, for which there are currently no guidelines for a healthy patient population. So the ability of health-care providers to compare patient reports to an established range of physiological CGM metrics from individuals without diabetes will be incredibly useful.

For individuals with diabetes, CGM metrics are also increasingly relevant both to clinical practice and research methodologies, including the well-established glucose ranges, time in range (70-180 mg/dL) (5), and more recently, time in tight range (70-140 mg/dL) (5-7). Therefore, it is important to understand the physiological time spent in specific ranges of CGM glucose for individuals without diabetes, which could serve as targets for individuals with diabetes or prediabetes.

## Materials and Methods

### Study Population

Our study included grandchildren of the original Framingham Heart Study (FHS) cohort who were enrolled in the Third Generation (n = 4095), New Offspring Spouses (n = 103), or Omni 2 (n = 410) cohorts in 2002 (8). Our study included participants from these cohorts who attended the fourth examination from September 2022 to December 31, 2023 (n = 1699), and wore a Dexcom G6 Pro CGM sensor for at least 7 full days on their upper arm or abdomen, with an available glycated hemoglobin A_1c_ (HbA_1c_) and fasting glucose measurement (n = 1175, Supplemental Fig. S1) (9). Diabetes was defined using self-report (current diagnosis or history of diabetes), HbA_1c_ greater than or equal to 6.5% (≥48 mmol/mol), venous fasting glucose of 126 mg/dL or greater, or taking glucose-lowering medications at the time of their study visit (n = 152); prediabetes was defined if participants had either an HbA_1c_ of 5.7% to 6.4% (39-47 mmol/mol) and/or venous fasting glucose of 100 to 125 mg/dL (n = 463); and the remaining participants did not meet criteria for prediabetes or diabetes, and are described as having “normoglycemia” for this report (n = 560). All participants provided written informed consent, and the institutional review board at Boston University Medical Center approved the study protocols.

### Continuous Glucose Monitoring Measurement

When participants came to the FHS Research Center for their study visit, a research technician applied a single Dexcom G6 Pro CGM sensor (Dexcom Inc) to their upper arm or abdomen, in the blinded mode. Participants were asked to wear the sensor for up to 10 days and mail the CGM sensor and transmitter back to the study site after the wear period.

### Continuous Glucose Monitoring Data Cleaning Procedures

We removed each participant's first and last partial days of data, including the first 12 hours of wear. Days with less than 70% complete data (<200 of the possible 288 values) were also removed (totaling only 9 days from 7 participants combined). Subsequently, participants who did not wear the CGM sensor for 7 or more full days (midnight-to-midnight) were excluded from the data set. The maximum and minimum possible recorded values were 400 and 40 mg/dL, with “high” or “low” messages recorded for data falling outside that range (as displayed in Table 1). We imputed “high” values as 401 mg/dL and “low” values as 39 mg/dL. We did not clean our data further because CGM data reports that patients may bring to their health-care providers are not cleaned.

**Table 1.** Demographics and continuous glucose monitoring metrics by glycemic status (n = 1175), mean (SD), or n (%)

	Normoglycemic n = 560	Prediabetes n = 463	Diabetes n = 152
Age, y	58.8 (9.0)	61.6 (8.3)	64.7 (8.0)
Men, n (%)	201 (35.9%)	251 (54.2%)	84 (55.3%)
BMI	26.5 (4.8)	29.3 (5.1)	32.3 (5.5)
HbA_1c_, %	5.2 (0.3)	5.5 (0.3)	6.6 (1.3)
Venous fasting glucose, mg/dL	91.3 (5.6)	104.2 (7.5)	135.8 (44.3)
Race and ethnicity, n (%)			
Hispanic/Latino	16 (2.8%)	19 (4.1%)	6 (3.9%)
NH Asian	10 (1.8%)	12 (2.6%)	6 (3.9%)
NH Black	4 (0.7%)	9 (1.9%)	4 (2.6%)
NH White	522 (93.2%)	410 (88.6%)	134 (88.2%)
NH American Indian, Native Alaskan, Native Hawaiian, or Pacific Islander	1 (.2%)	2 (.4%)	0 (.0%)
NH Multiracial	4 (0.7%)	8 (1.7%)	1 (0.7%)
NH Unknown race	3 (0.5%)	3 (0.6%)	1 (0.7%)
CGM-derived glucose metrics			
* *Mean, mg/dL	114.5 (11.5)	123.1 (14.3)	159.4 (44.5)
* *Median (IQR), mg/dL	111.6 (103.8-119.3)	120.30 (111.7-127.7)	155.0 (126.7-171.0)
* *Range of maximums, mg/dL	(80-376)	(130-401)	(167-401)
* *Range of minimums, mg/dL	(39-104)	(39-128)	(39-227)
* *Participants with “high,” n (%)	0 (0%)	1 (.2%)	14 (9.3%)
* *Participants with “low,” n (%)	111 (19.9%)	56 (12.1%)	18 (11.9%)
CGM time spent in glucose ranges (mg/dL), %			
* *<54	0.19 (1.75)	0.11 (.81)	0.06 (.25)
* *54-70	0.72 (2.97)	0.52 (2.46)	0.41 (1.43)
* *70-140	86.8 (11.0)	76.9 (18.7)	42.4 (30.6)
* *140-180	11.0 (9.5)	19.3 (14.9)	29.8 (17.7)
* *>180	1.3 (1.8)	3.3 (5.2)	27.4 (29.0)
* *70-180	97.8 (4.9)	96.1 (5.9)	72.1 (28.7)
* *180-250	1.2 (1.7)	3.1 (4.9)	19.0 (17.5)
* *>250	0.03 (0.16)	0.11 (0.65)	8.33 (18.80)
Participants spending time in glucose range >180 mg/dL:			
* *>1% time, n (%)	208 (37.1%)	267 (57.7%)	134 (88.2%)
* *>2% time, n (%)	118 (21.1%)	193 (41.7%)	126 (82.9%)

Abbreviations: BMI, body mass index; CGM, continuous glucose monitoring; HbA_1c_, glycated hemoglobin A_1c_; IQR, interquartile range; NH, non-Hispanic.

### Glycated Hemoglobin A_1c_, Fasting Venous Plasma Glucose, and Covariates

Venous whole blood was collected in 15% EDTA tubes (Monoject Blood) from participants who fasted 10 hours overnight. An aliquot was taken for HbA_1c_ measurement (Tina-quant Hemoglobin A1c, Roche Diagnostics). Blood glucose was analyzed using a Roche cobas c311 (GLUC3 Glucose HK, Roche Diagnostics) almost immediately after isolating plasma by centrifugation at 2500*g* at 4 °C.

### Statistical Analysis

Mean and standard deviation (SD), median, and interquartile range (IQR), or frequency of participants with certain demographic factors and CGM metrics were reported, stratified by glycemic status, age, and obesity status. CGM metrics were also presented in histograms, by glycemic status.

## Results

A total of 152 participants (12.9%) had diabetes, 463 participants (39.4%) had prediabetes, and 560 participants (47.7%) had normoglycemia. Normoglycemic participants had a mean age of 58.8 years, 64.1% were women, and 93.2% were non-Hispanic White (Table 1). Participants in the prediabetes and diabetes groups were older, 88% to 89% non-Hispanic White, and included higher proportions of men.

**Figure 1.**
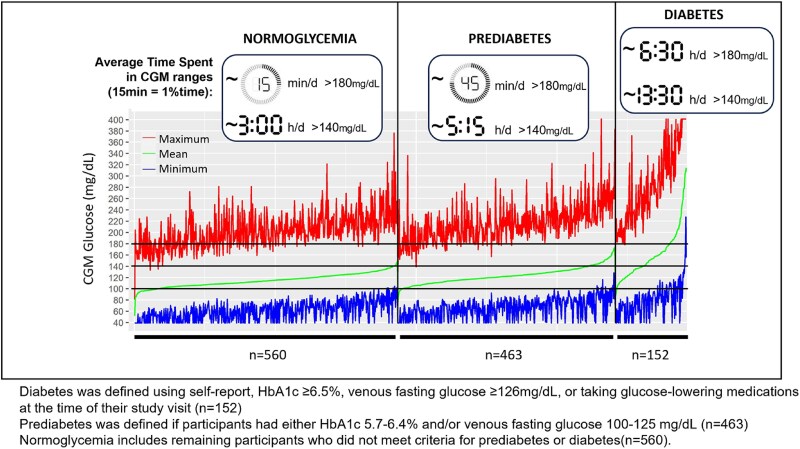
Ascending mean continuous glucose monitoring (CGM) glucose (middle line; green) for each participant within each glycemic status group (n = 1175). Maximum CGM glucose (top line; red) and minimum CGM glucose (bottom line; blue) also displayed.

**Figure 2.**
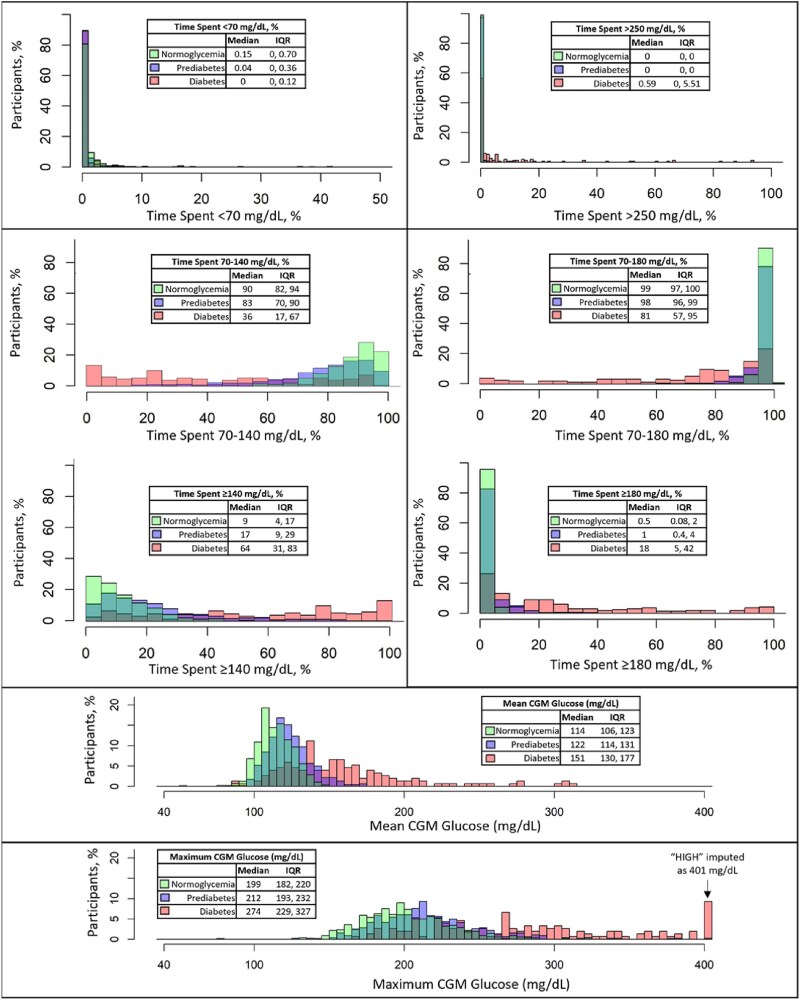
Histograms of continuous glucose monitoring (CGM) mean glucose, maximum glucose, and time spent in glucose ranges as a proportion of populations with normoglycemia, prediabetes, and diabetes (n = 1175). Histogram colors are partially transparent to show overlap.

All CGM metrics increased across glycemic status groups, further evidenced by increasing proportions of time spent in higher glucose ranges (see Table 1). Mean glucose had a similar distribution and was only 8.6 mg/dL higher in prediabetes compared to normoglycemia (123.1 vs 114.5 mg/dL); a majority of participants from both groups had a mean glucose between 100 and 140 mg/dL (Fig. 1). In normoglycemic participants, almost 98% of time was spent in the 70 to 180 mg/dL range, but less than 87% of time was in the 70 to 140 mg/dL range, with 11% of time at 140 to 180 mg/dL and 1.3% of time (>15 minutes/day) at greater than 180 mg/dL (see Table 1). Hence, normoglycemic participants (without elevated fasting venous glucose or HbA_1c_) spent approximately 3 hours/day (12.3% time) with CGM glucose at 140 mg/dL. Most normoglycemic participants had a maximum glucose level greater than 180 mg/dL, but rarely greater than 250 mg/dL (Fig. 2).

Normoglycemic individuals aged 60 years or older spent approximately 1% less time in the 70 to 180 mg/dL range compared to those younger than 60 years, with the additional time split between being spent above and below that range (Table 2). But there was a larger difference in CGM time in tight range (70-140 mg/dL) between nonobese individuals younger than 60 years (89.0% time) and nonobese individuals aged 60 or older (85.8%). Having obesity at either age was also associated with lower time spent in tight range, with older, obese adults having the lowest time spent in tight range, on average (83.7%), compared with other normoglycemic individuals. Unsurprisingly, participants with prediabetes and diabetes spent substantially less time in tight range (76.9% and 42.4%, respectively; see Table 1).

**Table 2.** Demographics and continuous glucose monitoring metrics for those with normoglycemia by age and obesity status (n = 560), mean (SD), or n (%)

	Middle age (<60 y)		Older age (≥60 y)	
	BMI <30 (n = 223)	BMI ≥30 (n = 61)	BMI <30 (n = 229)	BMI ≥30 (n = 47)
Age, y	51.9 (5.5)	50.6 (5.4)	66.4 (5.0)	65.5 (4.2)
Men, n (%)	85 (38.1%)	25 (41.0%)	77 (33.6%)	14 (29.8%)
BMI	24.7 (2.8)	33.6 (3.7)	24.8 (3.0)	34.5 (3.4)
HbA_1c_, %	5.1 (0.3)	5.2 (0.2)	5.2 (0.2)	5.3 (0.2)
Venous fasting glucose, mg/dL	90.0 (6.1)	92.6 (4.9)	92.0 (5.3)	92.3 (4.6)
Race and ethnicity, n (%)				
Hispanic/Latino	7 (3.1%)	2 (3.3%)	6 (2.5%)	1 (2.1%)
NH Asian	2 (0.9%)	1 (1.6%)	7 (3.1%)	0 (0.0%)
NH Black	2 (0.9%)	0 (0.0%)	2 (0.9%)	0 (0.0%)
NH White	211 (94.6%)	57 (93.4%)	209 (91.3%)	45 (95.7%)
NH American Indian, Native Alaskan, Native Hawaiian, or Pacific Islander	0 (0.0%)	0 (0.0%)	1 (0.4%)	0 (0.0%)
NH Multiracial	1 (0.4%)	1 (1.6%)	2 (0.9%)	0 (0.0%)
NH Unknown race	0 (0.0%)	0 (0.0%)	2 (0.9%)	1 (2.1%)
CGM-derived glucose metrics				
Mean, mg/dL	112.7 (10.7)	117.0 (11.3)	114.9 (11.8)	117.45 (13.3)
Median (IQR), mg/dL	109.9 (102.8-116.8)	115.0 (107.0-121.9)	111.6 (104.1-119.7)	115.0 (105.6-123.7)
Range of maximums, mg/dL	(148-288)	(142-308)	(80-376)	(135-307)
Range of minimums, mg/dL	(39-104)	(39-101)	(39-101)	(39-98)
* *Participants with “high,” n (%)	0 (0%)	0 (0%)	0 (0%)	0 (0%)
* *Participants with “low,” n (%)	44 (19.8%)	12 (19.6%)	48 (20.9%)	7 (14.8%)
CGM time spent in glucose ranges (mg/dL), %				
<54	0.11 (0.42)	0.07 (0.16)	0.32 (2.71)	0.06 (.10)
54-70	0.62 (1.31)	0.48 (.97)	0.94 (4.41)	0.50 (1.03)
70-140	89.0 (9.2)	85.3 (13.5)	85.8 (11.2)	83.7 (13.0)
140-180	9.3 (8.4)	13.0 (12.4)	11.4 (8.7)	14.4 (11.9)
>180	1.0 (1.5)	1.1 (1.8)	1.5 (1.9)	1.4 (2.4)
70-180	98.2 (2.1)	98.4 (2.0)	97.2 (7.1)	98.04 (2.4)
180-250	0.99 (1.48)	1.07 (1.69)	1.46 (1.76)	1.35 (2.20)
>250	0.02 (0.09)	0.03 (0.21)	0.04 (0.19)	0.05 (0.22)
Participants spending time in glucose range >180 mg/dL:				
>1% time, n (%)	62 (27.8%)	21 (34.4%)	108 (47.2%)	17 (36.2%)
>2% time, n (%)	37 (16.6%)	11 (18.0%)	62 (27.1%)	8 (17.0%)

Abbreviations: BMI, body mass index; CGM, continuous glucose monitoring; HbA_1c_, glycated hemoglobin A_1c_; IQR, interquartile range; NH, non-Hispanic.

Participants with prediabetes spent almost 20% of time in the 140 to 180 mg/dL range and more than 3% of time (>45 minutes/day) with CGM glucose at more than 180 mg/dL (see Table 1). Less than 25% of prediabetic participants reached a maximum CGM level greater than 250 mg/dL (see Fig. 2). Finally, those with diabetes spent the majority of time (>57%) at more than 140 mg/dL (see Table 1). Increasing proportions of the population across glycemic status categories (roughly 20%, 40%, and 80%) of those with normoglycemia, prediabetes, and diabetes spent more than 30 minutes/day (>2% time) with glucose at greater than 180 mg/dL (see Table 1).

## Discussion

Our results demonstrate that the physiologic range of CGM glucose in a cohort of middle-aged, non-Hispanic White individuals without diabetes or prediabetes is 70 to 180 mg/dL (∼97%-98% of their time). An additional 1% to 1.5% time (>15 minutes/day) is spent at greater than 180 mg/dL, but most do not reach 250 mg/dL. Comparatively, individuals with prediabetes spend roughly 30 minutes more time per day in the greater than 180 mg/dL glucose range and less than a quarter reach 250 mg/dL. Previous studies reported that normoglycemic adults spent between 93% to 97% of time, on average, in the 70 to 140 mg/dL range (3, 10). However, our results demonstrate that only 87% of time is spent in this “tight range,” varying by age and obesity status, and most of the remaining time (∼3 hours/day) CGM glucose is greater than 140 mg/dL. These data are relevant to the current debate about the potential value of time in tight range (70-140 mg/dL) as a glycemic target.

It is important for clinicians treating patients with diabetes to be aware that the physiologic CGM range among individuals with normoglycemia may include substantial time above 140 mg/dL and even acute periods above 180 mg/dL. Our observed proportion of time spent at more than 180 mg/dL was not shown in some smaller studies of normoglycemic adults (n < 100) wearing Dexcom G6 sensors by Shah et al (3) Abbott's Freestyle Libre Pro factory-calibrated sensors (10), nor in special populations of young pregnant women or athletes (11, 12). However, other studies have observed participants without diabetes achieving glucose levels greater than 180 mg/dL, using the Freestyle Libre and iPro2 (Medtronic) (13-16). Direct comparisons are difficult because these studies did not always report the average times spent in those ranges. Important differences in study design between our study and Shah et al may explain differing results despite using the same CGM sensor (3). Shah's study excluded individuals with obesity, and participants were relatively younger. Further, investigators directed participants to manually calibrate the (factory-calibrated) Dexcom G6 sensors, requiring participants to have access to their glucose levels (unblinded). In our study, participants wore the same sensors in a blinded mode without manual calibration. Although participants in both studies likely modified their behavior, as is true in any short-term observational study, blinding minimizes that behavior. Clinicians should expect that many patients without elevated HbA_1c_ or fasting glucose could have CGM reports showing glucose values greater than 180 mg/dL, but that their reports may differ by sensor and by the wearing protocol (eg, manual calibration). It is also possible that individuals experiencing substantial time greater than 180 mg/dL should not be viewed as “normal” physiologically; and certain glycemic patterns may be an early warning sign of metabolic and glycemic dysregulation that may be present in other clinical tests, such as an oral glucose tolerance test (17).

Our study design, integrating CGM sensor wear into an ongoing cohort study like the FHS, sets us up for answering important research questions, such as whether CGM metrics can predict the development of diabetes, as has been suggested in the A Estrada Glycation and Inflammation Study (AEGIS) in Spain of 499 individuals without diabetes (17). We will also be able to elucidate the determinants of CGM metrics/glycemic patterns among individuals with and without diabetes. Several studies have demonstrated that age, sex, body composition, diet, sleep, genetics, and gut microbiome are associated with CGM glucose metrics in individuals without diabetes (14, 18-21). The personalized responses to dietary composition trials (PREDICT), conducted in generally healthy individuals in the United Kingdom and United States, showed that genetics and the meal time of day explained the most interindividual variation in CGM postprandial glucose responses to standardized meals, compared to other predictors including age, sex, or body composition (14). In Israel, the 10 K study has reported that daily carbohydrate intake and visceral adipose tissue are also determinants of CGM measures of glycemic variability (18). Another interesting finding they reported was that body mass index appeared to be more strongly associated with the overall glycemic burden during sleep (measured by mean CGM glucose), but not the glycemic burden during waking hours. We hope to contribute to the understanding of these determinants of CGM patterns in subsequent analyses.

The strength of our CGM study lies in the nature of our participant pool, which largely comprised individuals without diabetes (n > 1000). As discussed previously, prior studies reporting on the physiological range of CGM levels across nondiabetic populations were much smaller, healthier, and included manual CGM sensor calibration by participants (3). Because we do not expect most individuals without diabetes to conduct manual sensor calibration while wearing the new CGM sensors that hit the wearables market this year (Abbott's Libre Lingo and Dexcom's Stelo) (1, 2), our study provides a more “real-world” design.

Our study was limited by a cross-sectional design, which cannot determine whether time spent in various glucose ranges among normoglycemic participants may be related to a higher risk of diabetes. We are also limited by lack of contemporaneous blood glucose measures, the use of only a single type of CGM sensor (Dexcom G6 Pro), and by the overrepresentation of non-Hispanic White individuals in our study population, who have been shown to have lower rates of dysglycemia compared to other races and ethnicities in the United States (22). Future studies will be needed to explore normative CGM metrics in more diverse study populations for increased generalizability. There are many important questions that can be answered in subsequent analyses, including assessing the influence of sex and menopausal status on glycemic patterns, such as potential hypoglycemic events (23). Another important future direction is standardizing CGM data-cleaning methods and identifying biologically implausible glucose values or patterns. Finally, exploring whether high glucose levels can be avoided among normoglycemic individuals with behavior modification will also be important.

In conclusion, time in tight range (70-140 mg/dL) has been growing in interest as a potential target for clinicians to help their patients with diabetes achieve better glucose control (5-7). However, our study provides evidence that individuals with normoglycemia spend approximately 3 hours/day above that tight range. The physiologic glucose ranges we report from a sample of more than 1000 individuals without diabetes may also be important for clinicians to reference as CGM sensors become more widely accessible to individuals without diabetes.

## Data Availability

Original data generated and analyzed during this study will be included in the following data repositories BioLINCC (https://biolincc.nhlbi.nih.gov/home/) and dbGap (https://www.ncbi.nlm.nih.gov/projects/gap/cgi-bin/study.cgi?study_id=phs000007.v33.p14) after data collection is complete.

## Abbreviations

CGM: continuous glucose monitoring
FHS: Framingham Heart Study
HbA_1c_: glycated hemoglobin A_1c_
IQR: interquartile range
SD: standard deviation
